# Enhancing the photocatalytic hydrogen production activity of BiVO_4_ [110] facets using oxygen vacancies†

**DOI:** 10.1039/d1ra07121a

**Published:** 2021-12-22

**Authors:** Jing Pan, Xiaoxue Ma, Wannian Zhang, Jingguo Hu

**Affiliations:** College of Physics Science and Technology, Yangzhou University Yangzhou 225002 China jghu@yzu.edu.cn

## Abstract

The activity of the hydrogen evolution reaction (HER) during photoelectrochemical (PEC) water-splitting is limited when using BiVO_4_ with an exposed [110] facet because the conduction band minimum is below the H^+^/H_2_O potential. Here, we enhance the photocatalytic hydrogen production activity through introducing an oxygen vacancy. Our first-principles calculations show that the oxygen vacancy can tune the band edge positions of the [110] facet, originating from an induced internal electric field related to geometry distortion and charge rearrangement. Furthermore, the induced electric field favors photogenerated electron–hole separation and the enhancement of atomic activity. More importantly, oxygen-vacancy-induced electronic states can increase the probability of photogenerated electron transitions, thus improving optical absorption. This study indicates that oxygen-defect engineering is an effective method for improving the photocatalytic activity when using PEC technology.

## Abstract 2

An oxygen-vacancy-induced internal electric field enhances the photocatalytic hydrogen production activity of a BiVO_4_ [110] facet.

## Graphical abstract

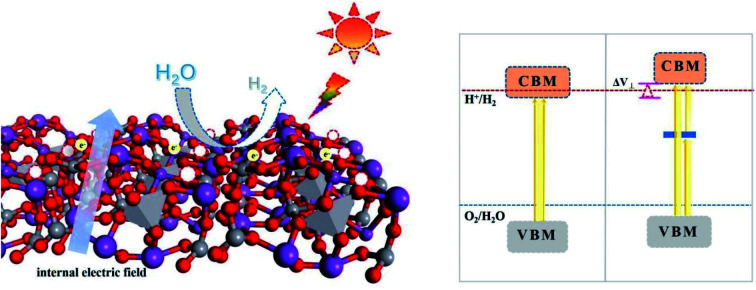

## 1. Introduction

Photoelectrochemical (PEC) water-splitting using solar energy to generate hydrogen is considered to be one of the more promising approaches for renewable energy production.^1,2^ Since the initial report of a TiO_2_-based photocatalyst, many semiconductors have been investigated, but the photocatalytic efficiencies are still low and far from being practically applicable because of the following problems: (1) weak visible-light adsorption due to wide band gaps; (2) fast electron–hole pair recombination; (3) low carrier mobility; and (4) band edge positions that do not match the water redox potentials.^3–5^ For an ideal photocatalyst, its valence band maximum (VBM) should be energetically lower than the O_2_/H_2_O potential and its conduction band minimum (CBM) should be higher than the H^+^/H_2_O potential; in addition, it should be active towards the H_2_ evolution reaction (HER) and O_2_ evolution reaction (OER).^6,7^ Recently, monoclinic clinobisvanite bismuth scheelite (ms-BiVO_4_) has attracted extensive attention due to its abundance, strong visible-light adsorption (with a direct band gap of 2.4 eV), and high activity for O_2_ evolution. For this photocatalyst, the VBM of BiVO_4_ is located at *ca.* 2.4 V *vs.* RHE, providing a sufficient overpotential for holes to photo-oxidize water. However, the CBM is below the H^+^/H_2_O potential, and the excited electrons cannot photo-reduce water.^8,9^ Additionally, poor carrier transport properties and rapid electron–hole recombination also limit the PEC performance.^10,11^ As a consequence, many measures have been taken, such as doping, morphology control, regulating different exposed facets, heterojunction construction, and surface decoration, to enhance its PEC activity.^12–15^ As is known, many examples of faceted BiVO_4_ polyhedra have been synthesized, and each exposed facet exhibits different thermodynamic and photocatalytic behavior; photogenerated electrons and holes can be preferentially separated and accumulated on [010] and [110] facets, where the [010] facet favors proton reduction and the [110] facet favors water oxidation.^16,17^ Zhao *et al.* realized a so-called hydrogen farm project *via* precisely tuning the (110)/(010) facets, achieving an overall solar-to-chemical efficiency of over 1.9% and a solar-to-hydrogen efficiency exceeding 1.8%. If the photocatalytic activity of a single (110) or (010) facet can be enhanced, the efficiency of photocatalytic hydrogen generation at a (110)/(010) facet heterojunction can also be enhanced. In this case, the oxygen reaction on the (110) facet is a complex reaction, thus, it is important to improve the photocatalytic activity of the (110) facet.^18^ Vacancy-defect engineering is a feasible method, utilizing electron redistribution and special chemical properties to enhance the photocatalytic activity.^19–21^

Recently, experimental studies have shown that oxygen vacancies (O_vac_) in a crystal structure could greatly improve the photocatalytic activity. For example, Zhao *et al.* reported the O_vac_-boosted photocatalytic nitrogen fixation of TiO_2_*via* providing more active sites with electron redistribution and enhanced electron transport.^22^ Our group have shown that surface O_vac_ supported charge separation and transfer, thus improving the OER performance of 3D nanoporous BiVO_4_.^23^ Although the use of O_vac_, which widely exist in metal oxides and are important for the PEC performance, is a promising strategy for enhancing the photocatalytic activity, the mechanism explaining the effects of O_vac_ on photocatalytic water-splitting remains contentious and poorly understood. In this work, we adopt the use of an O_vac_ to improve the photocatalytic hydrogen production activity of the BiVO_4_ [110] facet and investigate the mechanism *via* first-principles calculations. The calculated results show that the O_vac_ not only upshifts the band edge positions to satisfy the requirements of PEC water-splitting but it also assists photogenerated electron–hole separation and optical absorption, as a result of the O_vac_-induced electric field.

## 2. Computational model and methods

**Fig. 1:**
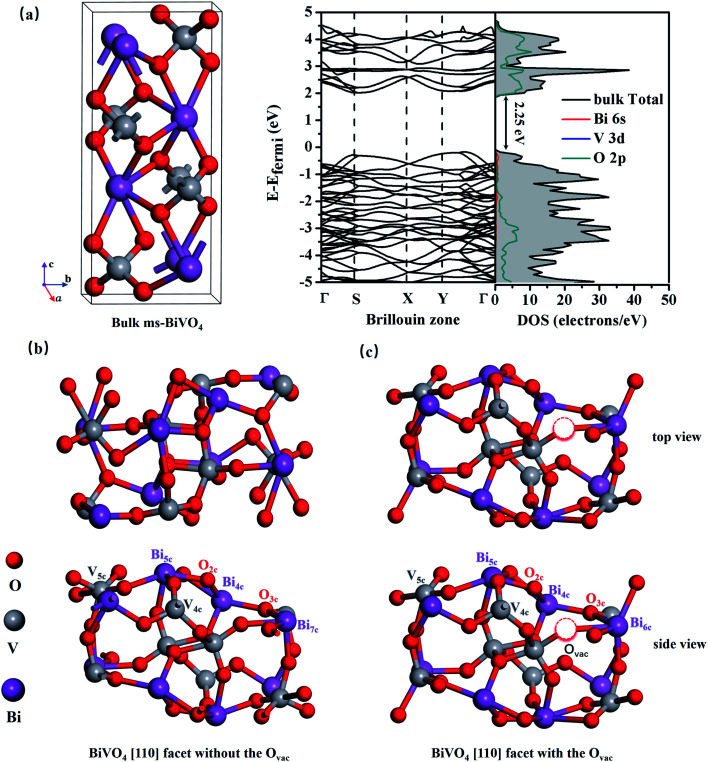
(a) The atomic structure and band structure of bulk ms-BiVO_4_. Top and side views of BiVO_4_ [110] facets (b) without an O_vac_ and (c) with an O_vac_. There are 8 Bi (purple), 8 V (gray), and 32 O (red) atoms in the BiVO_4_ [110] facet.

All calculations were based on the Vienna *ab initio* simulation package (VASP) with density functional theory (DFT),^24,25^ and the generalized gradient approximation (GGA) of the Perdew–Burke–Ernzerhof (PBE) functional was adopted.^26,27^ As shown in Fig. 1(b) and (c), supercells with a size of 11.9 × 7.3 × 23.5 Å^3^ containing 48 atoms were used to model bilayer BiVO_4_ with and without oxygen vacancies, and the models were constructed from the [110] surface of optimized bulk monoclinic BiVO_4_ with a 12 Å vacuum slab along the *z*-direction (see Fig. 1(a)). A plane wave cutoff energy of 400 eV and a total change in energy of 1.0 × 10^−5^ eV for geometrical optimization were employed, and the maximum stress was less than 0.01 eV Å^−1^. Monkhorst–Pack *k*-point grids of 5 × 5 × 1 for geometric optimization and 7 × 7 × 1 for electronic structure calculations were sampled as the Brillouin zones.

## 3. Results and discussion

As shown in Fig. 1(a), bulk BiVO_4_ is a layered monoclinic scheelite-phase structure containing BiO_8_ dodecahedra and VO_4_ tetrahedra, which are linked *via* Bi^3+^–O^2−^–V^5+^ connections and stacked along the main [001] axis direction with an interplanar distance of 2.97 Å (close to the experimental value of 2.89 Å).^28^ Bulk BiVO_4_ is a semiconductor, and the calculated band gap is 2.25 eV, basically in accordance with the experimental band gap of 2.40 eV. Additionally, the PBE-calculated lattice constants are *a* = 5.04 Å, *b* = 5.27 Å, and *c* = 11.89 Å, which are consistent with the experimental values of *a* = 5.09 Å, *b* = 5.20 Å, and *c* = 11.70 Å,^29^ confirming the reliability of the PBE method.

**Fig. 2:**
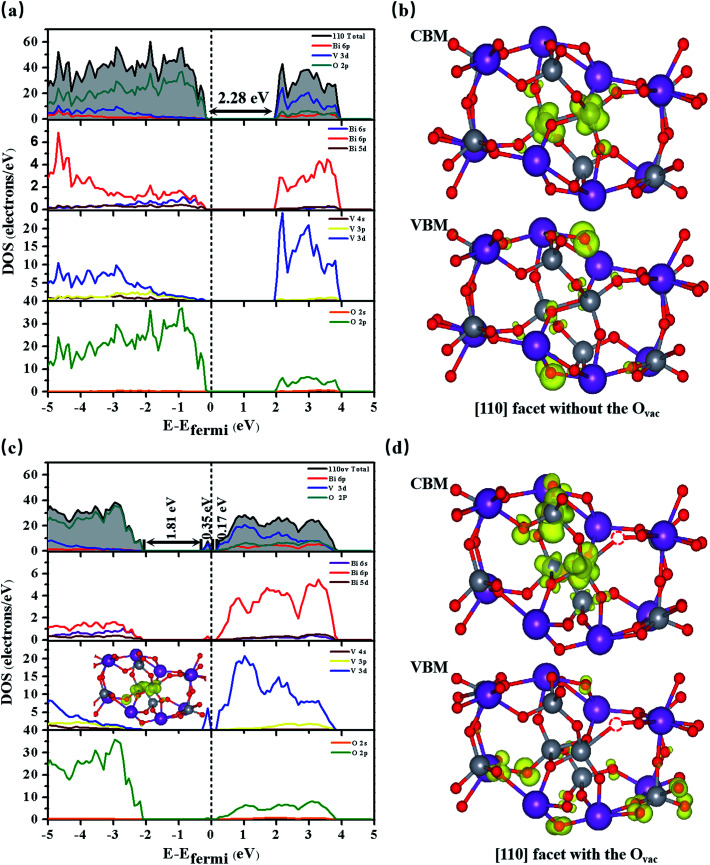
The total and partial densities of states (DOSs) of BiVO_4_ [110] facets (a) without the O_vac_ and (c) with the O_vac_; the inset shows partial charge density analysis of the resonance peaks. The charge density analysis of the VBM and CBM of BiVO_4_ [110] facets (b) without the O_vac_ and (d) with the O_vac_. The Fermi level is set to zero.

As shown in Fig. 1(b), the BiVO_4_ [110] facet is made up of 7-, 5-, and 4-coordinated Bi, 5- and 4-coordinated V, and 2- and 3-coordinated O; it retains semiconductor behavior with a band gap of 2.28 eV, and the VBM mainly consists of O 2p while the CBM is primarily composed of Bi 6p, O 2p, and V 3d (see Fig. 2(a)).

We create an O_vac_ near Bi_7c_, which is considered as an active site, as can be judged from the adsorption energy of H_2_O molecules given in the ESI.†^30^ As shown in Fig. 1(c), near the O_vac_, 7-coordinated Bi changes to 6-coordinated Bi, and Bi and V atoms bond to neighboring O atoms with smaller bond lengths and angles compared with the pure [110] facet. For example, the Bi_1_–O_1_ bond length varies from 2.33 Å to 2.27 Å, the V_1_–O_1_ bond length varies from 1.78 Å to 1.72 Å, and the O_1_–Bi_1_–O_2_ angle changes from 134.96° to 122.06° (see Table 1). This change in structure will result in a change in the electronic structure of the [110] facet. As shown in Fig. 2(c), the energy of the VBM in the [110] facet with the O_vac_ is lower than that in pure [110], indicating that holes are easily excited and easier to separate from the bulk to the surface.^31^ When the Fermi level is set to zero, the CBM is at 1.93 eV for the pure [110] facet, while it moves to 0.16 eV for the [110] facet with the O_vac_; the Fermi level is nearer the CBM for the [110] facet with the O_vac_, indicating an increase in the electron concentration and the generation of n-type semiconductor behavior. Furthermore, localized states exist in the band gap, coming from hybridization between V 3d and O 2p states neighbouring the O_vac_, which can also be seen in the partial density analysis in the inset of Fig. 2(c). These O_vac_-induced electronic states are conducive to electron transition from the VBM to these states, with a gap of 1.81 eV, and subsequently to the CBM, with a gap of 0.17 eV, benefiting the optical absorption.

**Table 1:** Bond lengths and angles for a pure BiVO_4_ [110] facet and a [110] facet with an O_vac_

	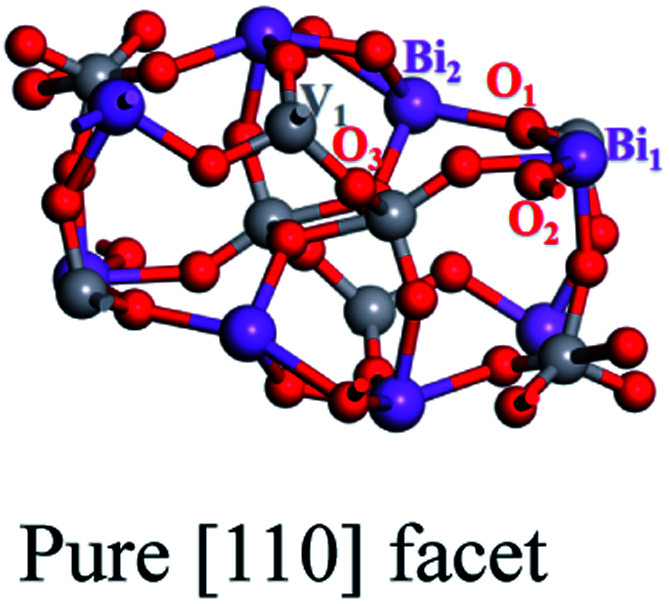	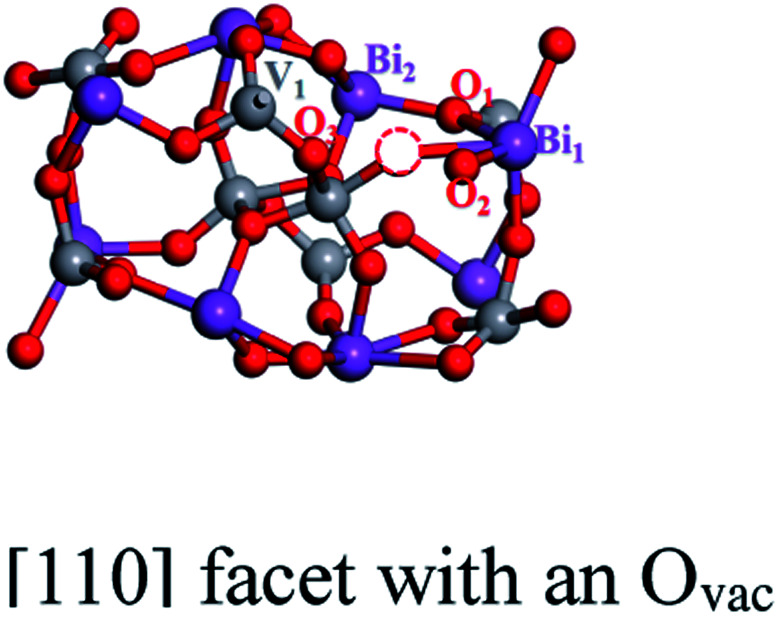
Bi_1_–O_1_	2.33 Å	2.27 Å
Bi_2_–O_2_	2.21 Å	2.20 Å
V_1_–O_1_	1.78 Å	1.72 Å
O_1_–Bi_1_–O_2_	134.96°	122.06°
O_1_–Bi_2_–O_3_	92.69°	91.59°

Comparing the partial charge densities of [110] facets with and without an O_vac_, as shown in Fig. 2(b) and (d), we find that charge in the VBM region is mainly concentrated on O atoms and charge in the CBM region is located mainly on V atoms for the pure [110] facet (see Fig. 2(b)). There are obvious changes in the [110] facet with the O_vac_: the charge in the VBM and CBM regions greatly increases because unmatched electrons and dangling bonds are present, favoring the catalytic behavior. Specially, the charge in the CBM region is distributed more on O atoms but still in the sublayer, the charge in the VBM moves to the surface, and the increased distance between the VBM and CBM is beneficial for electron–hole separation. Furthermore, we quantitatively analyse the electric dipole moments of BiVO_4_; an internal electric field is present with a magnitude of 13.01 D for the [110] facet with the O_vac_, which is associated with the greater geometry distortion and charge rearrangement and is stronger than that of the pure [110] facet (7.59 D). As is known, an induced electric field can effectively improve surface charge separation and change the photoelectrochemical impedance spectroscopy and transient absorption spectroscopy responses. For example, Zhang *et al.* have shown that tantalum doping induced an electric field in hematite homojunction nanorods, providing additional driving force to significantly improve charge separation both in the bulk and at the surface.^32^ Hussain *et al.* have shown that an oxygen-vacancy-induced internal electric field between [BiO]^+^ and [Br]^−^ had the remarkable capacity to assist effective charge separation and move charge to the surface from the bulk.^33^

**Fig. 3:**
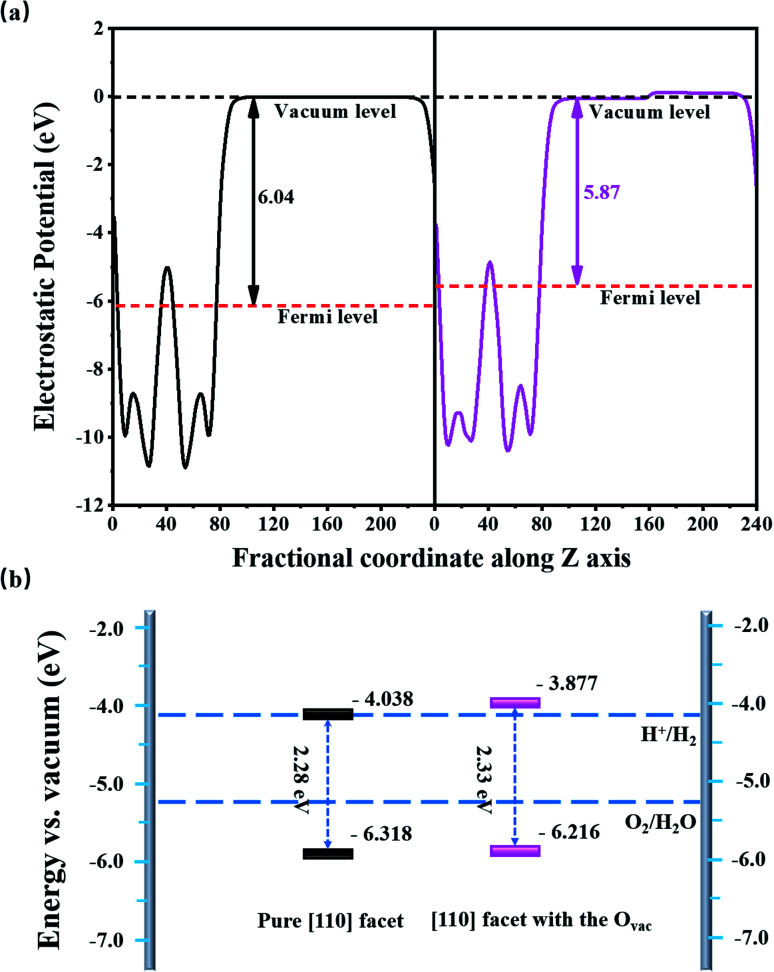
(a) The average electrostatic potentials along the *z* axis and (b) the band edge positions of BiVO_4_ [010] facets relative to the water redox potential with (pink) and without (black) the O_vac_.

Fig. 3(a) shows the average electrostatic potentials along the *z*-direction of the BiVO_4_ systems. The work function, defined as the difference between the vacuum level and the Fermi level, is 6.04 eV for the pure [110] facet, which is larger than that of the [110] facet with the O_vac_ (5.87 eV), indicating that charge is more easily transferred to the surface due to the existence of the O_vac_. Based on the electrostatic potential, the band edge energies (*e.g.*, the VBM and CBM) can be obtained *via* aligning the eigenvalues to the vacuum level.^34^ For the pure [110] facet, the VBM is −6.318 eV and the CBM is −4.038 eV, straddling the oxidation potential but not the H^+^/H_2_O potential; this means there is a lack of driving force for the HER, limiting the photocatalytic hydrogen generation abilities of BiVO_4_, which is in accordance with the experimental results.^35^ Compared to the pure [110] facet, the band edge positions of the [110] facet with the O_vac_ are upshifted by 0.161 eV; the VBM position is −6.216 eV and the CBM position is −3.877 eV, straddling the water redox region. The upshift mainly comes from the O_vac_-induced internal electric field caused by geometry distortion and charge rearrangement. As we know, the total dipole perpendicular to the surface component (*μ*_⊥_) causes the work function change, that is, Δ*W*_⊥_ = *μ*_⊥_/*Aε*_0_,^34,36^ where *A* and *ε*_0_ refer to the surface area of the unit cell and dielectric constant, respectively. Here, the dipole density is −0.428 D nm^−2^ and, therefore, the resultant work function change is −0.161 eV; based on Δ*V*_⊥_ = −Δ*W*_⊥_, the band edge upshifts by 0.161 eV, matching with the calculated energy shift based on the mean electrostatic potential.

**Fig. 4:**
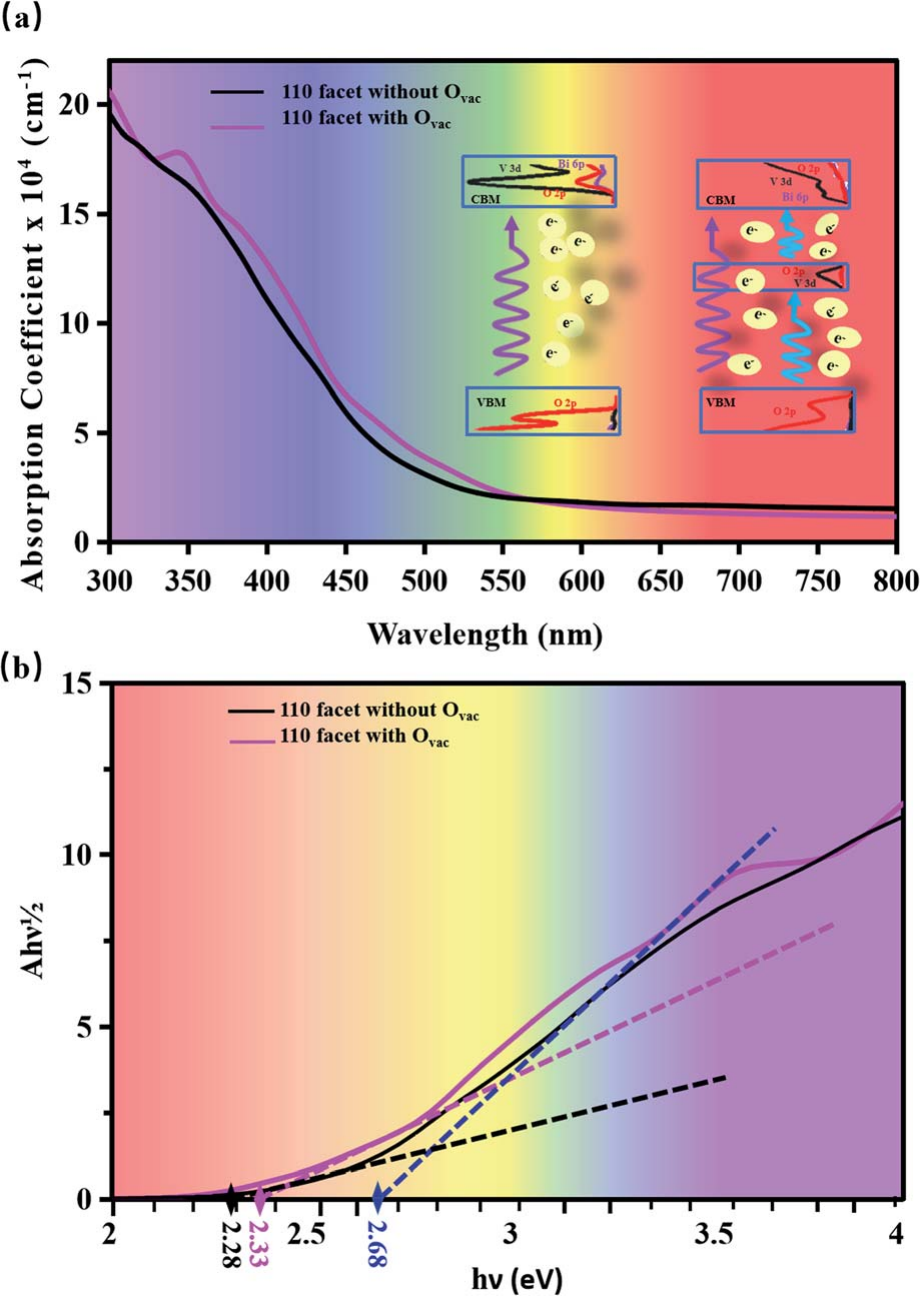
(a) Calculated absorption coefficients and (b) plots of (*Ahν*)^1/2^*vs.* photon energy for BiVO_4_ [110] facets with and without the O_vac_. The inset in (a) shows schematic diagrams of the electronic transitions without (left) and with (right) the O_vac_.

Fig. 4 displays the optical absorption of the BiVO_4_ [110] facets. We find that the absorption peak is at 196.4 nm for the pure [110] facet and at 197.7 nm for the [110] facet with the O_vac_. To clearly describe the change in band gap, we display (*Ahν*)^1/2^ as a function of *hν* in Fig. 4(b). The intercept of a tangent line to the first peak with the *x*-axis relates to the band gap. The intercept is 2.28 eV for the pure [110] facet, relating to the band gap. Compared to the pure [110] facet, two peaks appear for the [110] facet with the O_vac_: the intercept of the first peak relates to a band gap of 2.33 eV and the intercept of the second peak gives a band gap of 2.68 eV. The difference between the intercept of the first peak and the second small peak is 0.35 eV, relating to the energy range of local states due to the introduction of the O_vac_ [see Fig. 2(c)]. An enhancement of the optical absorption can be observed between 300 and 700 nm in the case of the [110] facet with the O_vac_. In fact, the absorption spectrum is closely connected with the electron transitions between the conduction bands and the valence bands. For the pure [110] facet, optical adsorption is derived from electronic inter-band transitions from O 2p at the VBM to O 2p, Bi 6p, and V 3d at the CBM (see the PDOS in Fig. 2(a)). For the [110] facet with the O_vac_, a peak appears at 350 nm, relating to an energy of 3.54 eV; the corresponding energy in the PDOS is 1.48 eV, as seen in Fig. 2(c), and, compared to the pure [110] facet, some electronic states appear at 1.48 eV due to the effects of the introduction of the O_vac_. Due to the presence of local states, the electron transitions include transitions not only from the host VBM but also from local states (*i.e.*, V 3d and O 2p) to the CBM. This indicates that the local electronic states not only favor electron transitions but they also enhance the transition probability.

Furthermore, the effects of the O_vac_ on the [110] facet have been explored *via* calculating its formation energy (*E*_form_ = *E*_O_vac__ − *E*_surf_ + ½*E*_O_2__, where *E*_O_vac__, *E*_surf_, and *E*_O_2__ are the total energies of BiVO_4_ [110] facets with and without the O_vac_ and molecular O_2_, respectively). The formation energy of the [110] facet with the O_vac_ is 3.86 eV, and this calculated result is comparable to what is reported in ref. 37, indicating that oxygen vacancies can easily be formed in the BiVO_4_ [110] facet. To further investigate the effects of the O_vac_ site and the O_vac_ concentration in the [110] facet, we create an O_vac_ neighbouring V_5c_ (see Fig. S2†), and create two and three O_vac_, forming O_vac_ concentrations of 6.25% and 9.38%, as shown in Fig. S3 in the ESI; †^30^ the calculated results show that the O_vac_ site and concentration have a great influence on the electronic structure and optical adsorption, thus affecting the photocatalytic properties.

## 4. Conclusions

Based on electronic structure calculations and band edge alignment analysis, we demonstrate that vacancy-defect engineering is a feasible strategy for improving the photocatalytic water-splitting activity of BiVO_4_. To this end, the O_vac_ plays an important role: (1) the O_vac_ excites the activity of neighbouring atoms due to unmatched electrons and dangling bonds; (2) the O_vac_-induced internal electric field is conducive to photogenerated electron–hole separation and can tune the band edges; and (3) the O_vac_-induced local electronic states favor electron transitions and enhance the optical absorption. As a result, the BiVO_4_ [110] facet can become a promising photocatalyst for water-splitting owing to the ideal band gap for enhanced optical absorption, the reduced electron–hole recombination, and the suitable band edges for water redox.

## Conflicts of interest

There are no conflicts to declare.

## Supplementary Material

RA-012-D1RA07121A-s001
