# Supplementary material for: Enhancing the photocatalytic hydrogen production activity of BiVO4 [110] facets using oxygen vacancies
Source: RSC Adv. 2021 Dec 22;12(1):540–5. doi: 10.1039/d1ra07121a (PMC8978636; doi:10.1039/d1ra07121a)
Supplement: RA-012-D1RA07121A-s001 [file RA-012-D1RA07121A-s001.pdf]

Electronic Supplementary Material (ESI) for RSC Advances.
This journal is © The Royal Society of Chemistry 2021

Supplementary Information

Enhancing Photocatalytic Hydrogen Production Activity in $BiVO_4$ [110] Facet by Oxygen Vacancies

Jing Pan, xiaoxue Ma, Wannian Zhang, Jingguo Hu

The site of the O_{vac} in $BiVO_4$ [110] facet

To investigate the site of the O_{vac}, we firstly investigate the photocatalytic active site, as shown in the Fig. S1(a), the H_2O molecule is adsorbed on surface different Bi atoms, the adsorption energies, defined as $E_{ads} = E_{tot} - E_{surf} - E_{H2O}$ (in which E_{tot}, E_{surf}, E_{H2O} are total energies of $BiVO_4$ [110] facets adsorbed with and without H_2O, and the energy of H_2O molecular), are -0.60, -0.36, -0.46 and -0.67 eV, the lower adsorption energy indicates more stronger binding, thus Bi4 is considered as the optimum adsorption site. Additionally, there are two O_{vac} could be created neighbouring Bi4, labeled by O_{vac1} and O_{vac2} in Fig. S2(b), the calculated formation energy E_{form} ($E_{form} = E_{Ovac} - E_{surf} + 1/2\ E_{O2}$, where E_{Ovac}, E_{surf} and E_{O2} are the total energies of $BiVO_4$ [110] facets with and without the O_{vac} and molecular O_2, respectively) is 3.86 eV for $BiOV_4$ with O_{vac1}, higher than that of $BiVO_4$ with O_{vac2} of 3.47 eV, indicating $BiOV_4$ with O_{vac1} was more stable than $BiOV_4$ with O_{vac2}.

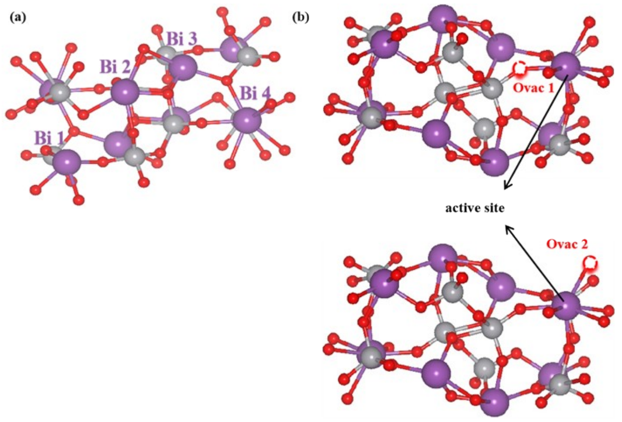

Fig. S1. Atomic structure of $BiVO_4$ [110] facets labeling (a) surface different Bi atoms for H_2O molecule adsorption and (b) two different O_{vac} with the O_{vac1} and the O_{vac2}.

The effect of the O_{vac} site on $BiVO_4$ [110] facet

We further create the O_{vac} coordinated with V_{5C} as shown in Fig. S2 (b). Compared to the O_{vac} near Bi_{7C}, the band gap decreases to 2.13 eV, there exist located states mainly consisted by Bi 6p, V 3d in the band gap. And the optical adsorption also changes accordingly, indicating the site of the O_{vac} has great influence on band gap, absorption spectra and density of states.

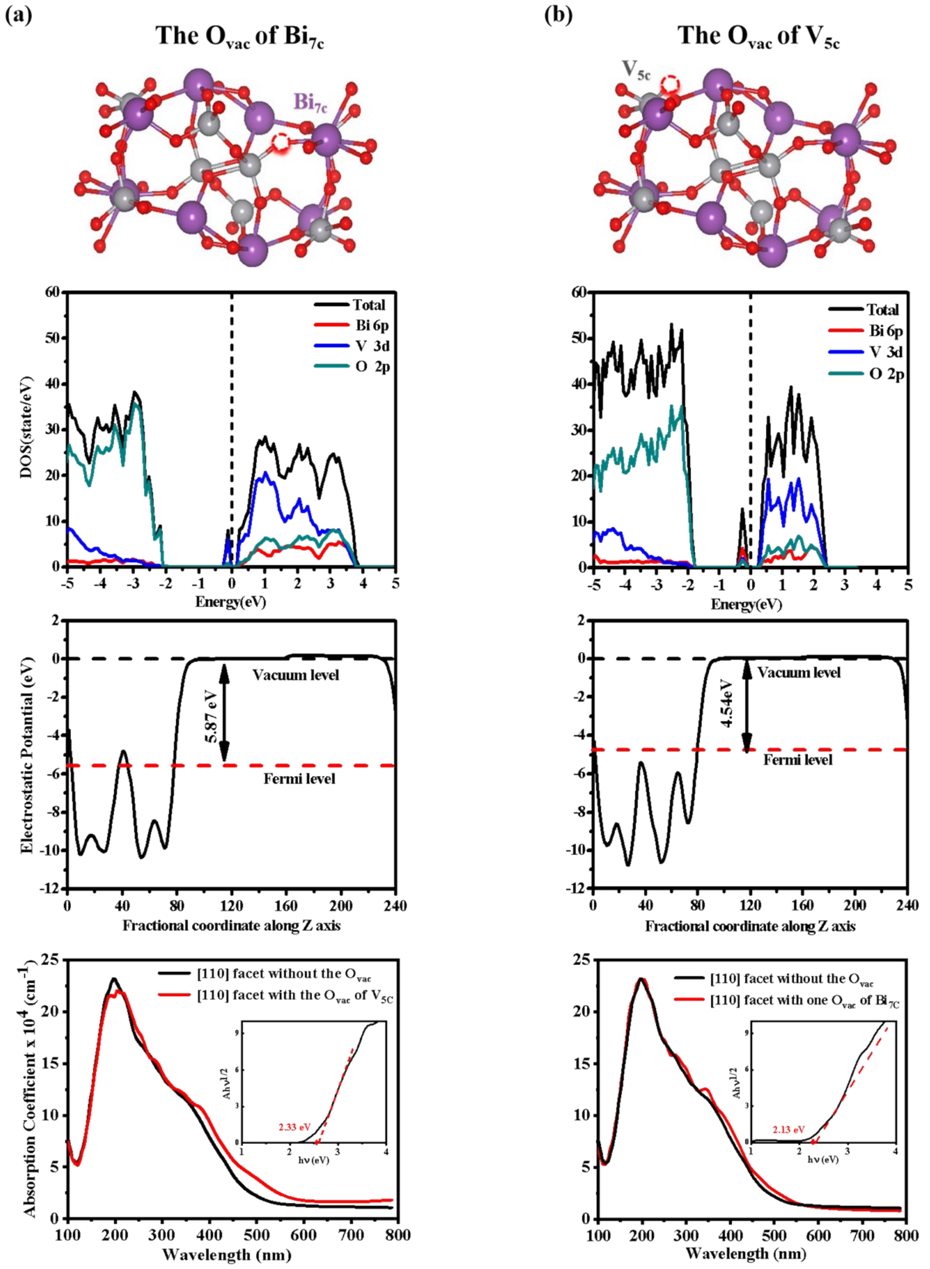

Fig. S2. The atomic structure, density of states, work function and absorption spectra of $BiVO_4$ [110] facets with (a) Bi_{7C} and (b) V_{5C}.

The effect of the O_{vac} concentration on $BiVO_4$ [110] facet

To investigate the effect of the O_{vac} concentration on [110] facet, we create one, two and three O_{vac} forming the concentration of 3.13%, 6.25 % and 9.38 % as shown in Fig. S3, the work function and optical adsorption change greatly. For [110] facet with two O_{vac}, the band gap increases to 2.32 eV, there exist higher located states mainly consisted by V 3d, O 2p in the band gap. And the optical adsorption improves slightly over 400 nm. While for [110] facet with three O_{vac}, the band gap decreases to 2.03 eV, there exist more located states mainly consisted by Bi 6p, V 3d, O 2p in the band gap. And the optical adsorption improves more slightly over 400 nm. Thus, the O_{vac} concentration has great influence on $BiVO_4$ [110] facet.

Additionally, due to the introduction of the O_{vac}, the band edges upshift and satisfy the requirement of the photocatalytic water-splitting. Thirdly, the work function decreases with the increasing of the O_{vac} concentration, indicating the charges more easily transfer from the bulk to the surface. Lastly, there appear more dangling bonds and the atom activity will be enhanced with the increasing of the O_{vac} concentration. Thus, we predicate that the the photocatalytic properties of [110] facet will be improved with the increasing of the O_{vac} concentration.

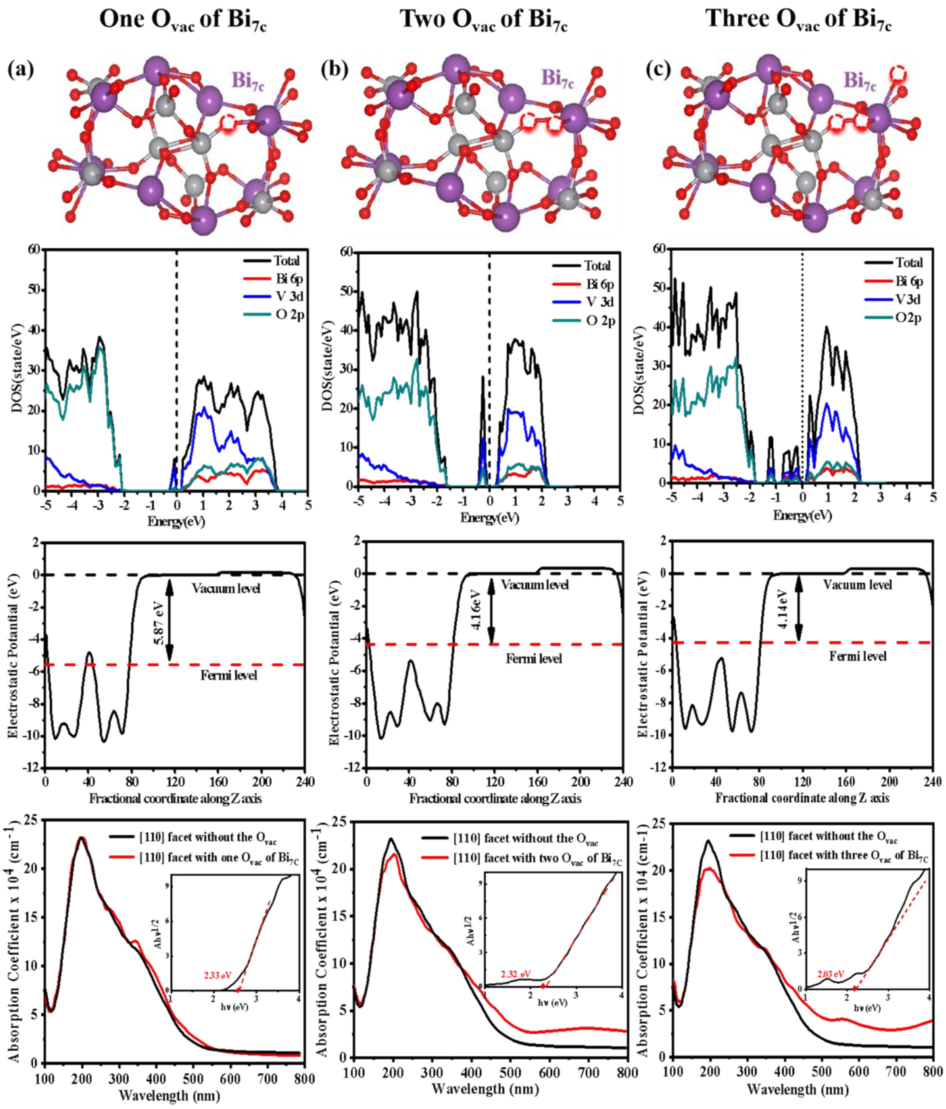

Fig. S3. The atomic structure, density of states, work function and absorption spectra of $BiVO_4$ [110] facets with the concentrations of (a)3.13%, 6.25 % and (c) 9.38 % of O_{vac}, the inset is optical band gap.
